# Evaluating the performance of multivariate indicators of resilience loss

**DOI:** 10.1038/s41598-021-87839-y

**Published:** 2021-04-28

**Authors:** Els Weinans, Rick Quax, Egbert H. van Nes, Ingrid A. van de Leemput

**Affiliations:** 1 Department of Environmental Sciences, Wageningen University, Wageningen, The Netherlands; 2 Computational Science, University of Amsterdam, Amsterdam, The Netherlands

**Keywords:** Systems biology, Environmental sciences, Mathematics and computing

## Abstract

Various complex systems, such as the climate, ecosystems, and physical and mental health can show large shifts in response to small changes in their environment. These ‘tipping points’ are notoriously hard to predict based on trends. However, in the past 20 years several indicators pointing to a loss of resilience have been developed. These indicators use fluctuations in time series to detect critical slowing down preceding a tipping point. Most of the existing indicators are based on models of one-dimensional systems. However, complex systems generally consist of multiple interacting entities. Moreover, because of technological developments and wearables, multivariate time series are becoming increasingly available in different fields of science. In order to apply the framework of resilience indicators to multivariate time series, various extensions have been proposed. Not all multivariate indicators have been tested for the same types of systems and therefore a systematic comparison between the methods is lacking. Here, we evaluate the performance of the different multivariate indicators of resilience loss in different scenarios. We show that there is not one method outperforming the others. Instead, which method is best to use depends on the type of scenario the system is subject to. We propose a set of guidelines to help future users choose which multivariate indicator of resilience is best to use for their particular system.

## Introduction

Some systems may show large transitions in response to very small changes in their environment. Such nonlinear responses have been documented in systems from various seemingly unrelated fields of study, including algae coverage in shallow lakes^1^, self-reported emotions of persons^2^, abundance of fish^3^, climatic variables such as ice cover^4^, or illness of animals or human beings^5,6^. Often these observed transitions are argued to be shifts from one stable state into another one, and these shifts between two stable equilibria are the focus point of this study. Because of internal feedback mechanisms, reversing conditions to a pre-shift situation does not necessarily cause a shift back to the old state^7^. Sometimes the shift back to the preferred state might not be possible at all. The likelihood of such transitions to be triggered by a perturbation, or in more technical terms the size of the stability landscape, is called the resilience of the system^8^. Being able to indicate if a system is losing resilience is one fundamental goal of the research on critical transitions^9^. If interactions and feedbacks of a system are well understood, fully parameterized models can help to simulate transitions. However, most of the aforementioned examples are inherently so complicated that accurate models do not exist. As an alternative to models, there are data driven methods that need time series data as input and that can provide a signal when a system is approaching a tipping point (the point where a shift to the alternative state is inevitable). The most well-known indicators of resilience loss are an increase in temporal autocorrelation (most often lag-1 autocorrelation)^10,11^ and an increase in variance^12^. These indicators are based on the phenomenon of critical slowing down. If a system approaches a tipping point, it will become intrinsically slower, such that recovery rate of disturbances decreases^9^. Indicators of critical slowing down have been applied to various lab-experiments^13,14^ and have been observed in real life systems^15,16^.

One limitation of the resilience indicators is that the theoretical framework used for their development is generally based on one-dimensional systems. It is therefore not clear if it is one-to-one applicable to the complexities that may occur in multivariate systems^17^. Multivariate systems, or network systems, are systems whose dynamics are described by multiple entities^18^. Examples include food webs of multiple interacting species, social networks where multiple individuals are observed, or spatial systems where the different locations in space can be viewed as different variables. Obtaining resilience indicators from time series of multivariate systems is fundamentally different from resilience indicators in univariate systems for two reasons. First, for most systems, it is not possible to obtain a quality time series of all variables, so a measurable subset should be chosen. Sometimes, the variables of interest are not measurable at all and a proxy is used (i.e. self-reported levels of emotions to track a persons mood in psychological studies^19^, or isotope measurements of $\delta ^{18}O$ in a sediment core to get an indication of past temperatures^20^). Second, for multivariate systems, recovery trajectories depend on which nodes are perturbed^21^. In mathematical terms it is said that multivariate systems may not have smooth potentials or that they are ‘non-gradient’. It has been suggested that non-gradient behaviour gives a dramatic boost for the possibilities in a system’s dynamics that are completely overlooked by the more traditional analyses^22,23^. One example of such behaviour of systems without a smooth potential is reactive behaviour, in which a perturbation leads to an initial response away from a stable equilibrium and only later recovers to it’s equilibrium position^24^. Reactivity has been proposed to be an intermediary step between stable systems and unstable systems and therefore might have properties of both^25^.

Despite this worry about the applicability, several multivariate indicators of resilience have been proposed. These can be divided into indicators based on univariate measures and multivariate indicators. A straightforward option using commonly used univariate measures is to choose one ‘representative’ variable to track over time^26^. However, this immediately leads to the problem of deciding which variable to measure. Another approach is to use the mean or median of commonly used univariate resilience indicators, such as autocorrelation and variance, for all variables^27,28^. However, the average value of all variables might be influenced by outliers, and does not exploit the full amount of information that is available in the multivariate signal. Among univariate indicators, autocorrelation is considered the more direct indicator of resilience which is more robust to noise, whereas variance is easier to measure and less sensitive to varying time intervals between consecutive data points^29^. A nonlinear alternative to autocorrelation is mutual information^30^.

Proposed multivariate indicators consist of a first step of dimension reduction technique, followed by the 1-D framework on the newly created 1-D data. This has led to the development of for example degenerate fingerprinting^11^, that calculates the autocorrelation of the data projection on the first principal component of a Principal Component Analysis (PCA). The advantage is that these techniques offer some new properties that in turn have been suggested as multivariate indicators of resilience loss, such as the explained variance of a PCA analysis^31^, the eigenvalue of a Min/Max Autocorrelation Factor (MAF) analysis^32^, the maximum value of the covariance matrix^33,34^, and the cross-correlation between individual elements^2,35^. Multivariate extensions to mutual information are for example information dissipation length (IDL)^36^ or information dissipation time (IDT)^37^ which measures how long a signal remains in the system before the information is lost. A possible disadvantage of these multivariate metrics is that they are data-hungry and that their theoretical foundation and link to critical transitions is not as well-developed as the more simple metrics. The multivariate indicators of resilience loss that we investigate in this study are listed in Table 1.

**Table 1:** Multivariate indicators of resilience loss used in this study.

Indicator	Description	Average based	Autocorrelation based	Variance based	Dimension reduction technique
Degenerate fingerprinting^11^	The autocorrelation of the data projection on the first principal component of a PCA	✗	✓	✓	✓
MAF autocorrelation^32^	The autocorrelation of the data projection on the first MAF. Alternatively his measure can be seen as the maximum autocorrelation in the system	✗	✓	✗	✓
MAF eigenvalue^32^	The minimum eigenvalue of a MAF analysis, calculated as the eigenvalues of the covariance matrix of the first difference of an SDS-transform of the original data	✗	✓	✗	✓
Mutual information^36^	Mutual information of time series with lagged time series of itself	✗	✓	✗	✗
Average autocorrelation	Autocorrelation averaged over all variables	✓	✓	✗	✗
Node maximum autocorrelation^26^	The autocorrelation of the variable with the highest autocorrelation	✗	✓	✗	✗
MAF variance^32^	The variance of the data projection on the first MAF	✗	✓	✓	✓
Node maximum variance^26^	The variance of the variable with the highest variance	✗	✗	✓	✗
Average variance	Variance averaged over all variables	✓	✗	✓	✗
PCA variance^11^	The variance of the data projection of the first principal component of a PCA. Alternatively this measure can be seen as the maximum variance in the system	✗	✗	✓	✓
Maximum value of covariance matrix^33^	The maximum value of the covariance matrix	✗	✗	✓	✓
Explained variance^31^	The explained variance of a PCA based on the covariance matrix, calculated as the maximum eigenvalue of the covariance matrix divided by the sum of all eigenvalues of the covariance matrix	✗	✗	✓	✓
Average absolute cross-correlation^2^	The average of the absolute values of all possible cross-correlations between variables	✓	✗	✗	✗

The set of recent indicators clearly reflects the interest in multivariate resilience indicators and the promising new opportunities. However they also pose new questions. Can we expect the multivariate indicators of resilience loss to accurately indicate an upcoming tipping point? What type of data do the different methods require? And can all the proposed indicators deal with the above mentioned multivariate data issues? Since most indicators have not yet been systematically compared (although some have^33^), we here evaluate the performance of the indicators from Table 1. The model we use to generate the time series is well-known plant-pollinator interaction model^31,38^. This model has been suggested to also be applicable to other bipartite networks where both facilitation and competition play a role. We chose to use this model, because it can be tuned to display the different types of dynamics that we explore here and it is simple enough to be representative of many systems that can undergo a critical transition such as a fold- or transcritical bifurcation. These bifurcations are part of a group of ‘zero-eigenvalue bifurcations’ where at the bifurcation point one eigenvalue of the systems Jacobian Matrix is zero.

We use the plant–pollinator interaction model to generate data where we know the true outcome which allows us to evaluate the performance of the different proposed indicators. We investigate the effect of six scenarios on the performance of the different indicators. These scenarios are meant as an illustration of some common data issues and system issues that can demonstrate the pros and cons of the list of indicators that we investigate, but obviously do not represent all issues that may be encountered when dealing with time series data. Four scenarios are associated with data acquisition: (1) limited data length, (2) limited data resolution, (3) observational noise/measurement noise, and (4) multiplicative noise as an example of a complex noise regime. The other two scenarios are associated explicitly with multivariate systems: (5) an incomplete set of observed variables and (6) reactivity as an example of non-smooth potential behaviour. The six scenarios are summarized in Table 2. All scenarios (except reactivity) are tested on a 4-dimensional (4D) version of the model and on two 20-dimensional (20D) models, of which one undergoes a full network collapse (all pollinator species are affected by the shift, all species go extinct), and the other approaches a partial network collapse (only half of the pollinators are directly affected by the shift, half of the pollinators go extinct, the other half of the pollinators and all plants remain alive). For reactivity, we only use the 4D model, since only this model can be tuned to display reactive behaviour.

**Table 2:** Scenarios used in this study.

Scenario	Description
Basic model	Time series have a length of 10,000 with a resolution of 0.1 steps. Gaussian white noise is implemented as a Wiener process with a standard deviation of 0.02. There is no measurement noise. All variables are taken into account.
Data length	Time series have a length of 1000 with a resolution of 0.1 steps. Gaussian white noise is implemented as a Wiener process with a standard deviation of 0.02. There is no measurement noise. All variables are taken into account.
Data resolution	Time series have a length of 10,000 with a resolution of 100 steps. Gaussian white noise is implemented as a Wiener process with a standard deviation of 0.02. There is no measurement noise. All variables are taken into account.
Measurement noise	Time series have a length of 10,000 with a resolution of 0.1 steps. Gaussian white noise is implemented as a Wiener process with a standard deviation of 0.02. Afterwards, a random number drawn from a normal distribution with 0 mean and a standard deviation of 0.08 is added to every datapoint in the time series. All variables are taken into account.
Multiplicative noise	Time series have a length of 10,000 with a resolution of 0.1 steps. Gaussian white noise is implemented as a Wiener process with a standard deviation of 0.02 multiplied by the value of the variable at that moment in time. There is no measurement noise. All variables are observed.
Subset variables	Time series have a length of 10,000 with a resolution of 0.1 steps. Gaussian white noise is implemented as a Wiener process with a standard deviation of 0.02. There is no measurement noise. All possible subsets of half of the variables are analyzed and their Kendall tau correlation is calculated. The 5% (worst case) and 95% (best case) percentiles of these correlations are depicted as the performance.
Reactive system	Same as basic model, but model parameters are chosen in such a way that the equilibrium in which the system resides is reactive.

## Results

**Figure 1:**
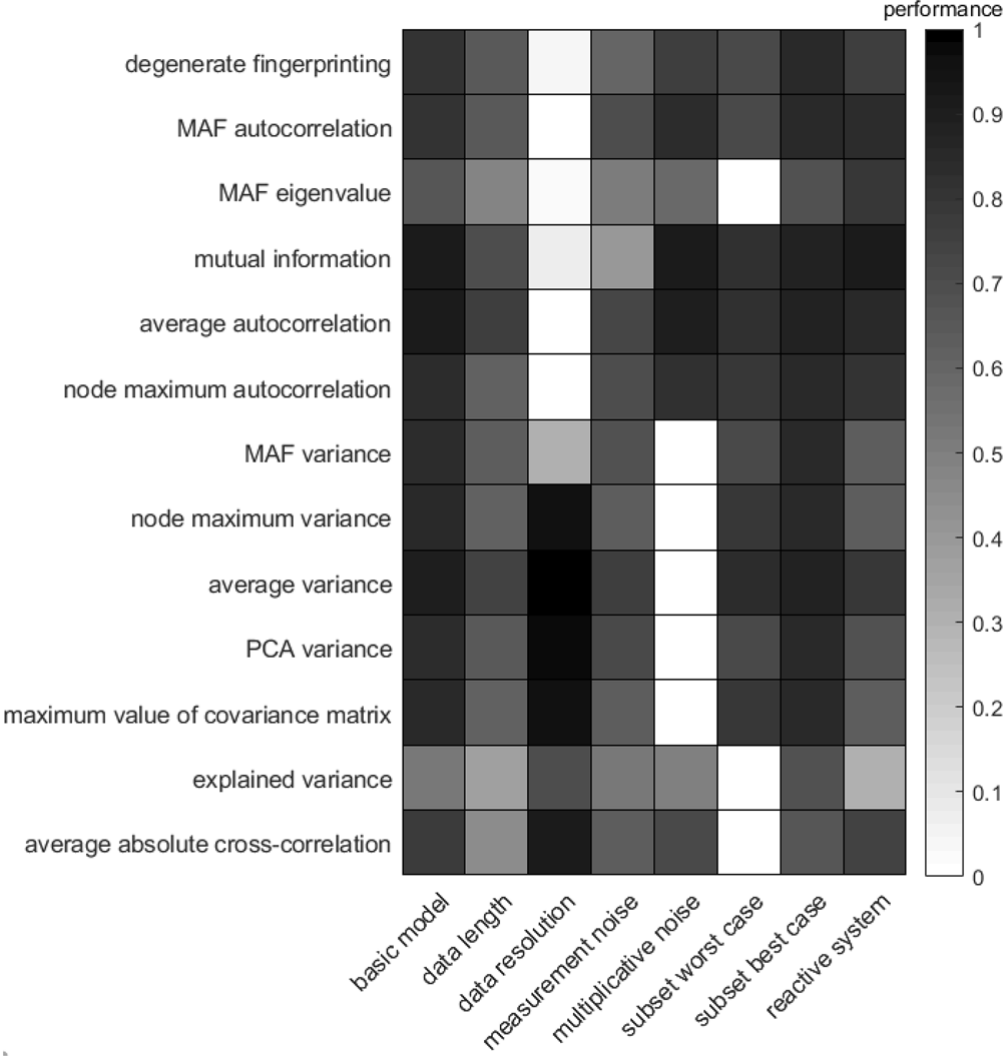
Performance of all indicators for different scenarios based on simulations with the 4D plant–pollinator model. Performance is calculated as the Kendall tau correlation of the change in indicator as the system approaches a critical transition.

**Figure 2:**
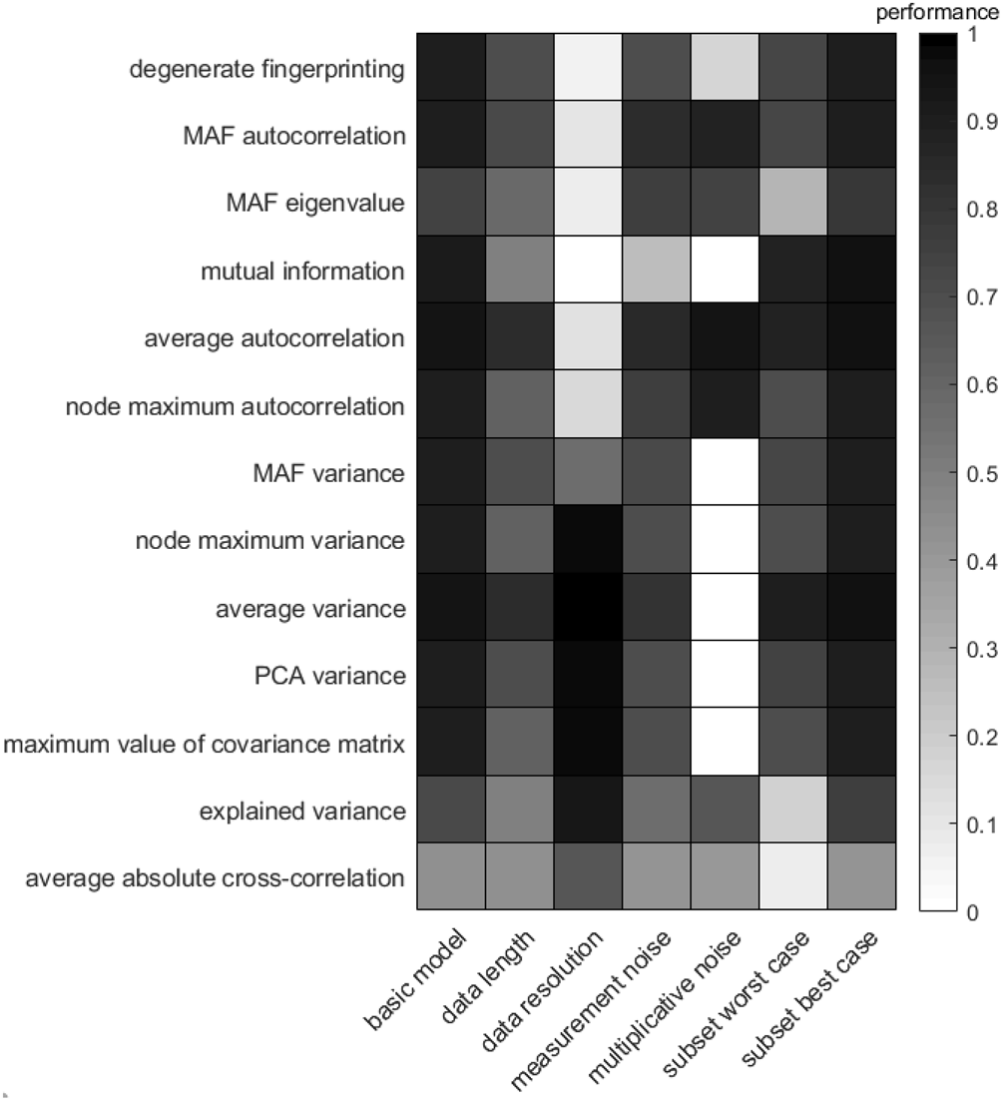
Performance of all indicators for different scenarios based on simulations with the 20D plant–pollinator model with a full network collapse. Performance is calculated as the Kendall tau correlation of the change in indicator as the system approaches a critical transition.

**Figure 3:**
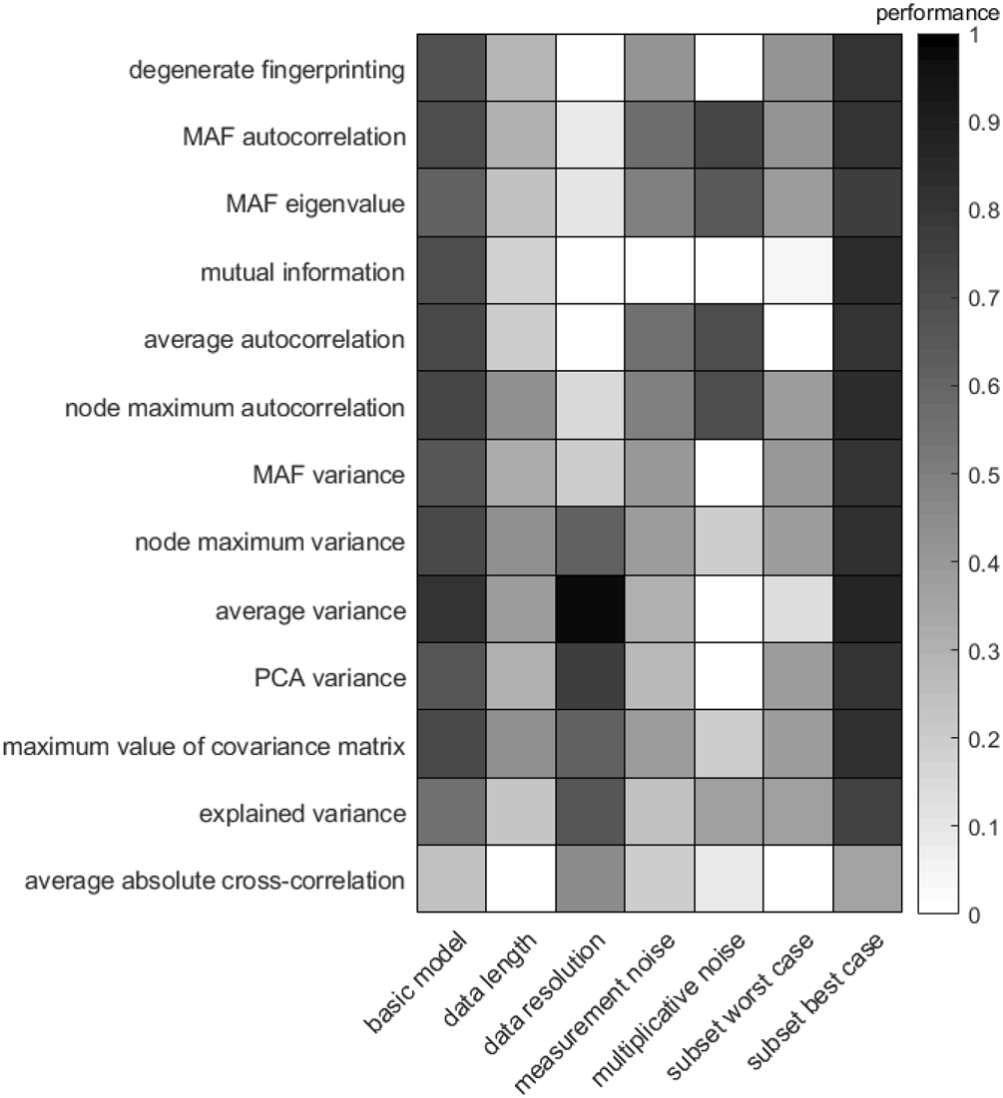
Performance of all indicators for different scenarios based on simulations with the 20D plant–pollinator model with a partial network collapse. Performance is calculated as the Kendall tau correlation of the change in indicator as the system approaches a critical transition.

The performance of the indicators based on the 4D model are summarized in Fig. 1. The results of the 20D model with a full network collapse are summarized in Fig. 2, and of the 20D model with a partial network collapse in Fig. 3. The performance of each indicator per scenario is measured as the Kendall tau correlation between the indicator itself and the value of the bifurcation parameter $r^{(A)}$ (see “Methods” section). In the next subsections we will discuss the performance per scenario, so per column in these figures.

### Performance in the basic model

The basic model scenario is unlimited by data length, resolution and accuracy, it has the most simple noise regime, all variables are observed, and the system behaves like a gradient system locally. Therefore, it adheres to all assumptions needed to apply indicators of resilience loss. In this scenario, as expected, all indicators increase as the bifurcation parameter increases, reflected by a high Kendall tau correlation. An example of the change in the indicator values for the 4D model can be found in Supplementary Fig. 4. In the 4D model the lowest Kendall tau correlation is found for the MAF eigenvalue and the explained variance (Fig. 1). Additional analyses indicate that the Kendall tau correlation of all indicators has quite high statistical specificity, i.e. it is able to distinguish between time series of a system that moves towards a tipping point and time series of a system that does not move towards a tipping point (Supplementary Fig. 7).

For the 20D system, a weak Kendall tau correlation is found for the average absolute cross-correlation between variables (Figs. 2, 3). In the 20D model with a partial network collapse all indicators have a lower performance than in the 4D and the 20D models where the entire network collapses. This indicates that partial network collapses, which are quite common especially in highly dimensional systems, are harder to detect than full network collapses.

### Performance for reduced data length

The data length scenario shows that all indicators are negatively affected by a reduction of the data length (from 10,000 data points in the basic model to 1000 data points in this scenario) (Figs. 1, 2, 3, second column). An illustration of the performance for 10,000, 1000 and 100 time points is provided in Supplementary Figs. 4–6. The effect of a gradually increasing data length is visualized in Supplementary Fig. 8. Interestingly, for the 4D model, most indicators quickly converge when the time series length is increased from 10 to 1000 data points. For the average absolute cross-correlation, explained variance and MAF eigenvalue, convergence happens slower and these indicators still seem to not have converged for a data length of 10,000 time points (Supplementary Fig. 8).

The best performing indicators in the reduced data length scenario are average autocorrelation and average variance, both in the 4D (Fig. 1) and the 20D model with the full network collapse (Fig. 2). These indicators are closely followed by the dimension reduction techniques degenerate fingerprinting, autocorrelation of the data projection on the first MAF (MAF autocorrelation), the variance of the data projection on the first PC (PCA variance) and the variance on the first MAF (MAF variance). In the 20D model with the partial network collapse (Fig. 3), all indicators performed quite poorly in this scenario. The best indicators are the node with the maximum autocorrelation or maximum variance, the average variance and the maximum value of the covariance matrix. The already poorer performing indicators MAF eigenvalue, explained variance, and average absolute cross-correlation are still the least effective indicators in this scenario. Another important observation is that the mutual information performs quite well for the reduced data length scenario in the 4D model, even though this method is known to be data-hungry. As the dimensions increase in the 20D models, the mutual information suffers more from the reduced data length scenario than in the 4D situation. The mutual information approximates a joint distribution from the data, and therefore it requires more data as dimensions increases.

### Performance for reduced data resolution

In the scenario of reduced data resolution, all autocorrelation-based indicators perform poorly compared to the variance based indicators (Figs. 1, 2, 3). Even the variance on the first MAF, which is also variance-based, performs poorly here, probably because the direction of the first MAF is not meaningful when data resolution is low. An illustration of the effect of a decreasing data resolution for the 4D model is provided in Supplementary Fig. 9. Perhaps surprisingly, all variance based indicators perform even better in the low-resolution scenario than in the basic model scenario (Figs. 1, 2, 3). Furthermore, the autocorrelation-based indicators too seem to benefit from a small decrease in data resolution (Supplementary Fig. 9). This can be explained by the fact that we fixed the data length at 10,000 points. The decrease in data resolution in this scenario thus entails an increase in simulation time. The increase in performance indicates that a higher sampling rate does not always provide more accurate results than a lower sampling rate, if the data length remains unchanged. The average variance is the indicator that outperforms the others for all three models in this scenario, which is especially clear in the 20-dimension model with a partial network collapse (Fig. 3).

### Performance for reduced data accuracy

A reduction in data accuracy affects the performance of all indicators negatively. The effect of a gradually increasing amount of measurement noise (or observational noise) is visualized in Supplementary Fig. 10. The least affected indicators are the autocorrelation on the first MAF, the average autocorrelation and the average variance. The mutual information is most negatively affected by the increase in measurement noise. The mutual information is the only indicator that does not make any assumptions on the distribution of the data, and therefore might have trouble estimating the distribution when the signal becomes more noisy.

### Performance for multiplicative noise

When the noise is modelled in a multiplicative way, all autocorrelation-based indicators maintain their high performance in the 4D model (Fig. 1). the mutual information and average autocorrelation are even completely unaffected by this change in noise type. In both 20D models however (Figs. 2, 3), the mutual information fails to detect the upcoming critical transition. In this scenario noise depends on the abundances of the variables and therefore the total noise changes over time. This might affect the bias in the mutual information, something we did not correct for in this study. The variance-based indicators, with the exception of the explained variance, are unable to detect an upcoming critical transition in this scenario, both in the 4D and 20D models. This is an interesting observation, since the explained variance is closely related to the maximum value of the covariance matrix. The advantage that the explained variance might have here, is that it is relative to the other values in the covariance matrix and is therefore less affected than the maximum value of the covariance matrix by unevenly distributed noise.

### Performance for reduced number of observed variables

The effect of only sampling a subset of the involved variables in the system depends on the type of collapse. Our reported performance levels reflect the 5% (worst case) and 95% (best case) quantile. In the case of a full network collapse we find that the MAF eigenvalue, the explained variance and the average absolute cross-correlation have the lowest performance (Figs. 1, 2, column ‘subset worst case’). For the 4D model, the other indicators work as good as in the basic model. For the 20D model however, all indicators are compromised, although the average autocorrelation, average variance, and mutual information are least affected by this scenario. In the case of a partial network collapse however, the performance of all indicators is highly reduced (Fig. 3, column ‘subset worst case’). The worst performance is found in the mutual information and the average-based indicators. Surprisingly, the best case scenario here even outperforms the basic model (Fig. 3, ‘subset best case’). Additional analyses of the distribution of the Kendall tau correlations show a large variation in performance (Supplementary Figs. 11–13). The average absolute cross-correlation does not detect a signal on average, but all other indicators have a high probability of detecting the upcoming shift (Supplementary Fig. 13). The high performances in these distributions correspond to situations where all or most variables that are affected by the bifurcation parameter are observed. The low performances appear for situations where the collapsed variables are not part of the observed subset (Supplementary Fig. 14). Interestingly, the increased performance in the best case column compared to the basic model, suggests that all indicators are hindered by the inclusion of variables that are not taking part in the shift.

### Performance for reactive systems

In the 4D version of our model, we tuned the parameters in such a way that the system is reactive. This did not substantially affect the performance of the indicators, other than that the average absolute cross-correlation performed slightly better in this scenario. Also the MAF-based indicators seemed to slightly improve their performance in this reactive system (Fig. 1).

Box 1: guidelines for choosing a multivariate indicator of resilience Based on our findings, we recommend the following guidelines to help deciding which indicator to use.

1. Before anything else, decide if the system you investigate could potentially be subject to a zero-eigenvalue bifurcation. Are there known processes that could cause a positive feedback loop? Have shifts been observed before? Is the system fluctuating around an equilibrium?
2. Check the autocorrelation of the included variables. If the autocorrelation is not significantly different from zero, this suggests the resolution of the data is too low to use autocorrelation-based indicators, so use a variance-based indicator instead.
3. Use system knowledge or take multiple measurements at the same time to determine how accurate the data is. If there is a low data accuracy (high observational or measurement noise), use a dimension-reduction technique or use the average autocorrelation or variance.
4. Use system knowledge or perform experiments to get an understanding of how the noise behaves. In real systems the noise is most likely a combination of observational/measurement noise and system noise. If the system noise varies over time, such as in our multiplicative noise scenario, use an autocorrelation-based indicator.
5. Irrespective of which method is chosen, we recommend to do a data suitability test^32^, to test whether the data is of sufficient length to reliably calculate the resilience indicators.

## Discussion

Our results show that in our basic model (Figs. 1, 2, 3, first columns) all proposed indicators rise preceding a critical transition (see also Supplementary Fig. 4). Furthermore, even though non-gradient behaviour has been described as a major issue for multivariate dynamical systems^23,24^, our results find no evidence that reactivity is a problem for the multivariate indicators of resilience tested for here (Fig. 1, last column). However, not all indicators perform well in every situation and our modelled scenarios help us to understand why certain resilience indicators may fail under particular circumstances.

In the scenario of reduced data length, all indicators have a lower performance than in the basic model. This can simply be explained by the fact that less data leads to weaker statistics.

In the reduced data resolution scenario all variance-based indicators and the absolute average cross-correlation remain strong, while all the autocorrelation-based indicators are weakened. If the sampling resolution is too low, the indicators directly capturing the speed of the system, i.e. based on autocorrelation, will fail to indicate critical slowing down. However, the variance-based indicators, which are essentially indirect measures of slowing down, are not affected by lower resolutions^29^. This is true for both univariate and multivariate timeseries. Whether data resolution is problematic depends on the sampling frequency and the actual speed of the system. For instance, in systems where the activity of taking measurements might affect the dynamics of the system, such as the questionnaires about the mood or behaviour of individuals^39^, obtaining data with high data length and high resolution can be challenging. Also many ecosystems are difficult to sample on a sufficiently high resolution and sufficiently long time scale, since interactions often occur on long time scales (order of magnitude of several years are not uncommon)^40^. However, long time series do exist in ecology^41^, and modern sensor techniques are proving their potential, in both aquatic ecology^42,43^ and vegetation studies^44^. Fields of science that more commonly gather data via automated monitoring devices, such as in medical applications (e.g. wearables), high resolution data is more abundant^45^.

Higher measurement noise leading to reduced data accuracy has a detrimental effect on all indicators. Mutual information is most strongly affected by reduced data accuracy in comparison to the other indicators. Mutual information is closely related to autocorrelation. It has for instance been shown that for a bivariate normal distribution the lag-1 autocorrelation and mutual information are directly linked to each other (with the relation $MI= -\frac{1}{2} log(1-\rho ^2)$, where $\rho$ is the lag-1 autocorrelation)^46^. Therefore, the difference in performance between the two has to be linked to data length. This links to our observation both in the noisy scenarios, as well as in the high dimensional systems, where mutual information needs an increasing amount of data to estimate the distribution of the data, since it is not assuming any predefined distribution. Autocorrelation has fewer degrees of freedom and is therefore less influenced by noisy data when data is limited. Our analysis was based on stationary time series of 10,000 time points for every value of the control parameter, which is considered quite a lot of data in most applications. Therefore mutual information might not be the most practical choice as an indicator of resilience unless the number of degrees of freedom can be significantly reduced or large amounts of data are available.

We simulated a complex noise scenario by replacing additive noise with multiplicative noise, meaning that the amount of noise depends on the variable level (here species abundance). The multiplicative noise scenario hardly affects the autocorrelation based indicators in the 4D model, but it has a detrimental effect on all variance-based indicators. In the 20D systems it also affects degenerate fingerprinting and mutual information. Degenerate fingerprinting is most likely affected because a PCA, which is a step in degenerate fingerprinting, is heavily influenced by changes in noise regimes. In our multiplicative noise scenario we multiplied the noise by the species abundances. Other ways to investigate complex noise regimes are by changing the way it is distributed over variables^32,47^ or by adding the noise to modeled parameters instead of variables^29^. Ecological systems are often most realistically modeled with noise added to a parameter (for example a simple system of bacteria where temperature affects the growth rate and in that way affects the abundance), and therefore in ecological systems autocorrelation-based indicators might be the more robust choice^29^.

Our findings suggest that the subset of variables that is looked at, can have a major influence on the results. This problem was first described for univariate analyses of three variables in a three-dimensional system^47^, and still appears for the multivariate methods that we investigate here. Importantly, not all variables in a multivariate system approaching a critical transition are subject to critical slowing down, so when only a subset is observed, it depends on the subset whether a loss of resilience will be signalled. In models with a full network collapse, several indicators hardly show a loss of resilience: the MAF eigenvalue, the explained variance and the average absolute cross-correlation (Figs. 1, 2). In contrast, in our 20D model where only a part of the variables collapses, all indicators have a reduced performance. Cross-correlation is most affected by not observing all variables (Supplementary Figs. 11–13). Some combinations of variables even give an indication of an increase in resilience as the system moves towards the tipping point (Supplementary Figs. 7–9). In this scenario, only 5 of the 20 species are directly affected by the changing environmental condition. The variables are all interacting with each other, so in principle this information could be distributed through the network. However, as we show, the signal is not picked up by all subsets of variables. The performance essentially depends on the fraction of observed variables that are part of the transition. Performance is high if all variables that are collapsing are observed, and performance reaches a minimum when none of the observed variables are collapsing (Supplementary Fig. 14). One important note is that in real systems it is typically unknown how many of the observed variables will be part of the shift (i.e. the location on the horizontal axis in Supplementary Fig. 14). The prevalence of this scenario for all real-world cases therefore suggests a cautious interpretation of all univariate and multivariate resilience analyses.

Our analysis of multiple indicators of resilience loss in multivariate timeseries shows that there is not one indicator that clearly outperforms the others. It depends on the scenario which indicator is favourable, similar to what was previously found for one-dimensional systems^48^. Some of the scenarios that we present here can be tested for, providing a user with an indication of the type of system they are dealing with. Box 1 provides some guidelines and questions to consider before choosing a multivariate indicator of resilience. Given the complexity of the matter, we are unable to propose a step-by-step flow chart that recommends one indicator based on a set of questions. However, we do believe that these considerations can guide the decision-making process.

**Figure 4:**
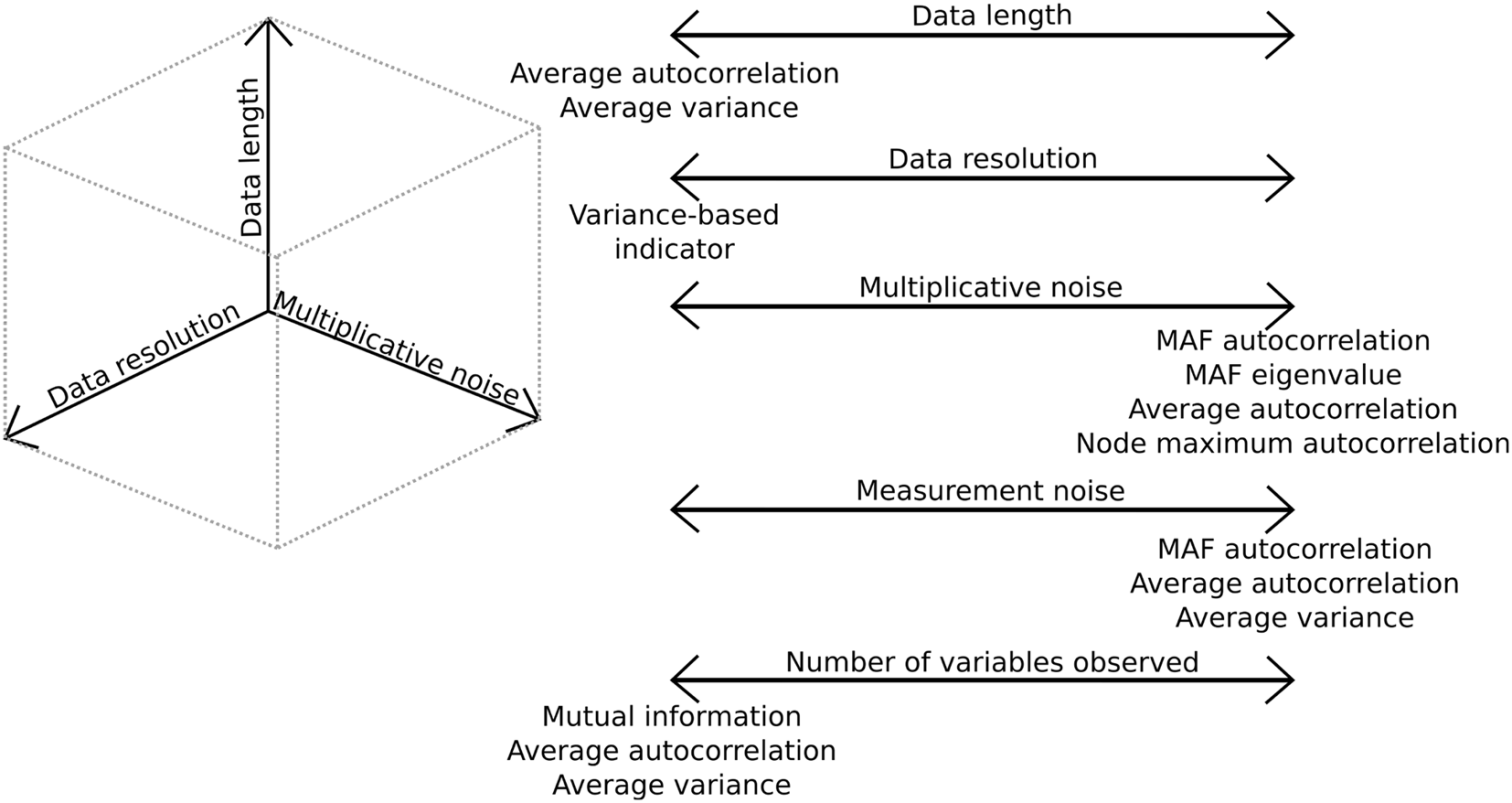
Conceptual image of the multivariate space a system can be in, of which we here visualize three axes. In reality, any issue (data issue such as data length or system issue such as reactivity) can be seen as an axis in this coordinate system, resulting in a high-dimensional space. Depending on the location of the system in this coordinate system, some indicators are preferable over others in terms of performance. For example, as the system under investigation moves to the low end of the ‘data resolution axis’, variance based indicators are the preferred choice, whereas on the high end there is no limitation on the choice of indicator. If the system is found on the low end of the ‘data length’ axis, average autocorrelation, average variance, or mutual information could be the best choice for an indicator. As a system moves along the ‘multiplicative noise’ axis, our analysis suggests that autocorrelation-based indicators, with the exception of mutual information, are the best choice. In our analysis we investigate the effect of moving along several axes of this conceptual coordinate system. The regions in between are yet to be explored.

It is important to realize that the scenarios we discussed generally do not happen in isolation. In order to fully map the performance of resilience indicators, ultimately a multidimensional coordinate system is needed where each of the scenarios that we used can be seen as one of the axes in the coordinate system (as illustrated in Fig. 4. This coordinate system has many additional axes that are not investigated here). In every region of this coordinate system some indicators might perform well, whereas others are unable to detect any change in resilience. Some regions might not be suitable for any (of the currently used) multivariate indicators of resilience loss. This figure illustrates one limitation of our study: our scenarios investigate what happens along each axis, whereas the spaces in between are left unstudied. For example in our 4D model, in every scenario we demonstrate that some indicators can correctly predict the upcoming tipping point. However, when the data has a low resolution, in combination with multiplicative noise, and only a subset of data is observed (not uncommon for empirical datasets), all indicators will likely have a low performance.

In this study, we focus on the performance of indicators for approaching zero-eigenvalue bifurcations as classic examples of critical transitions. Obviously, there are many other types of transitions that are out of the scope of this study. Consequently, the parameters in our model were tuned in such as a way that more complex bifurcations such as hopf bifurcations, or global bifurcations, are not taken into account. Also, we did not consider abrupt shifts caused by an increase in noise^49^, in which case flickering might be a more promising indicator than any of the indicators we have investigated here^50^. Also, some rapid transitions might be caused by rapid changes in the environment in which case no indicators of critical slowing down are expected^51,52^. Therefore our conclusions should only be seen in the context of slowly approaching fold or transcritical bifurcations, which is why the first guideline in Box 1 suggests to always first consider the type of transition that is expected before applying any indicator of resilience to the data.

Some multivariate indicators of resilience loss have been suggested to not only predict when a systems is losing resilience, but also which variables are most affected by it^27,32,34^ or even where the future state of the system can be found^31^. To answer these ‘follow-up questions’, MAF or PCA related indicators seem quite appropriate, since they provide a ‘direction’ (i.e. a combination of variables that are most affected by the changing conditions or that recover slowly when perturbed simultaneously) in addition to a resilience indicator. These are the indicators that fail in our ‘subset of variables’ scenario and therefore we do not recommend to use them to predict an upcoming tipping point. However, we do see a use for these indicators to answer questions such as “which variables are affected by an upcoming shift?” and “what will the future state look like?”^31,32^.

There is a group of indicators based on information theory that could potentially also be useful to predict upcoming transitions (such as Fisher information^53^ or entropy measures^54^). These indicators also infer properties of dynamical systems, but so far have not been applied to infer a systems resilience. Therefore, we did not include them in our analysis. We do touch upon information theoretical indicators by including the mutual information indicator. This indicator accurately points to a loss of resilience in the basic model, but it seems quite sensitive to the scenarios we have investigated here. Furthermore, some indicators not directly linked to critical slowing down have been found to precede critical transitions, such as critical fluctuations^55^. Future comparisons can take into account these, and other, extensions to the resilience indicators investigated here.

The idea that critical transitions are preceded by generic indicators that cross over multiple scientific domains is an exciting premise that has attracted justifiable widespread attention. However, even though the theoretical work has led to many successful discoveries, the application to multivariate empirical data remains challenging. Awareness of the potential pitfalls that can be encountered in real data and an understanding of their effect on the different indicators may help to interpret the growing body of resilience research.

## Methods

### Model

To investigate the effect of various multivariate data issues, we use a well-known simplistic model that we can tune to display a wide range of dynamics and which can be pushed towards a fold or a transcritical bifurcation (two types of critical transitions). The model has been used to describe plant–pollinator interactions^56^, but can be used to describe a wide range of phenomena where facilitation and competition both play a role^31^. The model has a deterministic part and a stochastic part. The deterministic part is used to calculate the dominant eigenvalue of the Jacobian (as the ‘true’ resilience of the system) and the eigenvalue of the corresponding Hermitian (as the reactivity of the system). The model is implemented in Grind for Matlab^57^ and integrated using an Euler-Maruyama scheme with an integration step of 0.01. The time unit is arbitrary. We sampled data points with an interval of 0.1 time point (so after 10 integration steps), unless stated otherwise.

1 $$\begin{aligned} dA_{k}= & {} \left[r^{(A)}_{k}A_{k}+\frac{\sum _{i=1}^{S_{P}}\gamma ^{(A)}_{ki}P_{i}}{1+h_{k}\sum _{i=1}^{S_{P}}\gamma ^{(A)}_{ki}P_{i}}A_{k}-\sum _{l=1}^{S_{A}}c^{(A)}_{kl}A_{l}A_{k}\right]dt+\sigma _{A_k}dW, \end{aligned}$$

2 $$\begin{aligned} dP_{i}= & {} \left[r^{(P)}_{i}P_{i}+\frac{\sum _{k=1}^{S_{A}}\gamma ^{(P)}_{ik}A_{k}}{1+h_{i}\sum _{k=1}^{S_{A}}\gamma ^{(P)}_{ik}A_{k}}P_{i}-\sum _{j=1}^{S_{P}}c^{(P)}_{ij}P_{j}P_{i}\right]dt+\sigma _{P_i}dW. \end{aligned}$$

In this model, $A_k$ represents the abundance of pollinator species *k* and $P_i$ represents the abundance of plant species *i*.

The parameter *r* describes the per capita growth rate, which in this case can be negative. The parameter $\gamma$ describes the mutualistic interactions with other species of the other group (where $\gamma ^{(P)}_{ik}$ stands for the positive effect that plant species *i* experiences from pollinator species *k*). In the model, a saturation is assumed for high abundances of mutualistic partners, where parameter *h* is the half-saturation constant. The parameter *c* is a competition term describing the negative effect that pollinators have on each other ($c^{(A)}_{kl}$) and on themselves ($c^{(A)}_{kk}$) and that plants have on each other ($c^{(P)}_{ij}$) and on themselves (*c*_*ii*_^*(P)*^). Consistent with previous work^56^, we assume that species can not out-compete each other in the absence of mutualistic partners, so each species has a higher competition with itself than with the other species.

First we use this model to simulate data with two plants ($S_P = 2$) and two pollinators ($S_A = 2$) with default parameters set as $r^{(P)} = [-0.5, -0.5]$ (non-reactive) or $r^{(P)} = [2.2, 2.2]$ (reactive), $\gamma _{11} = \gamma _{22} = 1$, $\gamma _{12} = \gamma _{21} = 0.8$ and $c_{11} = c_{22} = 0.3$, $c_{12} = c_{21} = 0.1$ for both plants and pollinators. The half saturation constant *h* is 0.5 for all species.

In this model, the relative growth rates between the plants and the pollinators can cause the system to become reactive or pass a tipping point. We find the combinations of parameter values of $r^{(P)}$ and $r^{(A)}$ for which the system undergoes a tipping point by calculating when the Jacobian matrix is zero (Supplementary Fig. 3). Additionally, we find the parametervalues where the system becomes reactive (Supplementary Fig. 3).

We find that the combination of parameter values for which the model becomes reactive and for which it passes a tipping point by setting the noise to zero and calculating the eigenvalues of the Jacobian and Hermitian matrix dependent on the values of $r^{(P)}$ and $r^{(A)}$ (Supplementary Fig. 1). The parameter $r^{(A)}$ is the bifurcation parameter and changes in the basic model from [− 0.3 − 0.2] to [− 0.68 − 0.58], in the reactive model from [− 0.91 − 0.81] to [− 1.45 − 1.35]. We tuned this bifurcation parameter such that the change in resilience for both scenarios is exactly the same: the dominant eigenvalue moves from − 0.45 to − 0.15 in both scenarios. In line with previous studies, in the reactive scenario, the system is not reactive in the beginning but it becomes reactive as the system moves towards the tipping point^25^ (in Supplementary Fig. 1 the dashed line at $r^{(P)}=2.2$ crosses the blue line indicating the system becomes reactive).

For our simulations, we increase $r^{(A)}$ stepwise in 50 steps. For every value of $r^{(A)}$ we generate stochastic time series by setting $\sigma$ to 0.02 for both the plants and the pollinators, unless stated otherwise.

Next, we use the same model to simulate data with ten plants ($S_P = 10$) and ten pollinators ($S_A = 10$). For this model, we could not use default parameters from other studies, so we did a random search to find parameters for which the system starts in an equilibrium where all species are present (abundance > 0.1) and that slowly moves either to a full network collapse (10 pollinators are affected by the changing parameter, all 20 species become extinct) or a partial network collapse (5 pollinators are affected by the changing parameter, those 5 species become extinct). An illustration of the behaviour of these 20D models can be found in Suppementary Figs. 1 (full network collapse) and 2 (partial network collapse). In line with the 4D model $h=0.5$, $c^{(P)}_{ii} = c^{(A)}_{kk} = 0.3$ and $\gamma ^{(A)}_{ii} = \gamma ^{(P)}_{kk}= 1$. Parameter settings for the other variables can be found in the Supplementary Materials Sections 1 and 2.

### Indicator performance

We use the generated data to calculate how the indicators in Table 1 change for different values of the bifurcation parameter. In line with previous work^34,48^, we calculate the Kendall tau correlation between the indicator and the value of the bifurcation parameter as a measure of how well the indicator performs. If the Kendall tau correlation was lower than zero, the performance was set at zero. The Kendall tau correlation is a rank correlation and therefore distinguishes if there is a trend. It provides no information on how this trend evolves over time (i.e. a linear trend can have the same correlation as an exponential trend).

All indicators with the exception of mutual information were implemented in Matlab. Mutual information^30^ was calculated in Python using the NPEET package^58^, using 3 neigbors for the kNN algorithm (k = 3), a base of 2 and no bias correction (alpha = 0).

We test for the sensitivity of the indicators to data length by repeating the tests for different lengths of data while keeping the sampling interval constant. A data length of 1000 was chosen for the reduced data length scenario. Next, we test for the sensitivity to data resolution by sampling every 100rd time point (instead of the default of 0.1). Last, we test for the sensitivity to accuracy of the data by applying artificial measurement noise (or observational noise) by adding random values from a normal distribution to the data with $\mu = 0$ and $\sigma$ ranging from 0 to 0.3. A $\sigma$ of 0.08 was chosen for the reduced data accuracy scenario. We create data with multiplicative noise by multiplying the Wiener process with the species abundance. We analyze the effect of not observing all variables by repeating the analysis for all possibilities of half of the variables, so for our 4D model this leads to 6 options (4 choose 2) and for the 20D model this leads to 184,765 options (20 choose 10). In this scenario we determine the performance as the 5% and 95% quantile of all Kendall tau correlations and label them ‘worst case’ and ‘best case’ respectively. Using the 4D model, we test for the effect of reactivity by generating data with the reactivity parameter set to its reactive value ($r^{(P)} = 2.2$) and repeating the same tests as described before. This was only done for the 4D model as the 20D model could not be made reactive while ensuring all species were present in the system. The scenarios, including a short description, are summarized in Table 2.

## Supplementary Information

Supplementary Information
