# Supplementary material for: Evaluating the performance of multivariate indicators of resilience loss
Source: Sci Rep. 2021 Apr 28;11:9148. doi: 10.1038/s41598-021-87839-y (PMC8080839; doi:10.1038/s41598-021-87839-y)
Supplement: Supplementary file 1 — Supplementary Information [file 41598_2021_87839_MOESM1_ESM.pdf]

Evaluating the performance of multivariate indicators of resilience loss - Supplementary Information.

Els Weinans, Rick Quax, Egbert H. van Nes & Ingrid A. van de Leemput

1 parameter settings of the 20D model with full network collapse

$c^{(A)} =$

$$\begin{bmatrix}
0.3000 & 0.0360 & 0.0462 & 0.0166 & 0.0365 & 0.0149 & 0.0536 & 0.0368 & 0 & 0.0221 \\
0.0220 & 0.3000 & 0.0242 & 0.0885 & 0.0292 & 0.0602 & 0.0371 & 0.0809 & 0.0229 & 0.0086 \\
0.0233 & 0.0763 & 0.3000 & 0.0547 & 0.0354 & 0.0228 & 0.0528 & 0 & 0.0549 & 0.0618 \\
0.0510 & 0.0637 & 0 & 0.3000 & 0.0355 & 0.0057 & 0.0087 & 0.0556 & 0.0185 & 0.0498 \\
0.0577 & 0.0251 & 0.0795 & 0.0500 & 0.3000 & 0.0534 & 0.0302 & 0.0537 & 0.0325 & 0.0523 \\
0.0707 & 0.0165 & 0.0128 & 0.0471 & 0.0492 & 0.3000 & 0.0224 & 0.0573 & 0.0351 & 0.0350 \\
0.0371 & 0.0774 & 0.0375 & 0.0434 & 0.0245 & 0.0150 & 0.3000 & 0.0771 & 0.0413 & 0.0834 \\
0.0516 & 0.0164 & 0.0183 & 0.0406 & 0.0463 & 0.0355 & 0.0307 & 0.3000 & 0.0507 & 0.0457 \\
0.0289 & 0.0318 & 0.0278 & 0.0602 & 0.0326 & 0.0403 & 0.0308 & 0.0332 & 0.3000 & 0.0306 \\
0.0551 & 0.0336 & 0.0267 & 0.0392 & 0.0454 & 0.0556 & 0.0248 & 0.0124 & 0.0488 & 0.3000
\end{bmatrix}$$

$c^{(P)} =$

$$\begin{bmatrix}
0.3000 & 0.0512 & 0.0299 & 0.0454 & 0.0033 & 0.0304 & 0.0392 & 0.0284 & 0.0382 & 0.0571 \\
0 & 0.3000 & 0.0264 & 0.0192 & 0.0393 & 0.0504 & 0.0755 & 0.0020 & 0.0708 & 0.0283 \\
0.0458 & 0.0463 & 0.3000 & 0.0394 & 0.0216 & 0.0335 & 0.0404 & 0.0209 & 0.0097 & 0.0329 \\
0.0400 & 0.0478 & 0.0414 & 0.3000 & 0.0348 & 0.0370 & 0.0022 & 0.0331 & 0.0321 & 0.0345 \\
0.0807 & 0.0348 & 0.0521 & 0.0429 & 0.3000 & 0.0675 & 0.0318 & 0.0622 & 0.0369 & 0.0425 \\
0.0516 & 0.0079 & 0.0439 & 0.0347 & 0.0460 & 0.3000 & 0.0768 & 0.0280 & 0.0779 & 0.0484 \\
0.0294 & 0.0456 & 0.0646 & 0.0001 & 0.0266 & 0.0385 & 0.3000 & 0.0769 & 0.0436 & 0.0576 \\
0.0522 & 0.0476 & 0.0355 & 0.0641 & 0.0255 & 0.0359 & 0.0339 & 0.3000 & 0.0523 & 0.0398 \\
0.0026 & 0.0699 & 0.0547 & 0.0246 & 0.0471 & 0.0396 & 0.0630 & 0.0355 & 0.3000 & 0.0591 \\
0.0320 & 0.0109 & 0.0470 & 0.0213 & 0.0489 & 0.0139 & 0.0517 & 0.0353 & 0.0298 & 0.3000
\end{bmatrix}$$

$\gamma^{(A)} =$

$$\begin{bmatrix}
1.0000 & 0.7256 & 0.8165 & 0.8032 & 0.8804 & 0.7901 & 0.8946 & 0.7545 & 0.9086 & 0.8047 \\
0.7243 & 1.0000 & 0.7728 & 0.7887 & 0.8128 & 0.8364 & 0.9992 & 0.8955 & 0.7779 & 0.8616 \\
0.8501 & 0.8354 & 1.0000 & 0.8472 & 0.7449 & 0.8142 & 0.6800 & 0.6601 & 0.9537 & 0.9296 \\
0.7165 & 0.8669 & 0.6222 & 1.0000 & 0.8427 & 0.8191 & 0.7604 & 0.6323 & 0.6300 & 0.7554 \\
0.8607 & 0.8280 & 0.5856 & 0.7303 & 1.0000 & 0.8277 & 0.8152 & 0.7924 & 0.9418 & 0.7169 \\
0.7751 & 0.8574 & 0.8164 & 1.0000 & 0.7621 & 1.0000 & 0.8343 & 0.7611 & 0.8105 & 0.7735 \\
0.8250 & 0.9945 & 0.8621 & 0.8314 & 0.8036 & 0.8109 & 1.0000 & 0.8486 & 0.7733 & 0.5809 \\
0.6731 & 0.7307 & 0.8968 & 0.7996 & 0.8268 & 0.6182 & 0.6832 & 1.0000 & 0.8913 & 0.5988 \\
0.8080 & 0.9226 & 0.9267 & 0.9080 & 0.8974 & 0.9306 & 0.6748 & 0.7757 & 1.0000 & 0.8893 \\
0.7584 & 0.7300 & 0.7736 & 0.9048 & 0.8394 & 0.8409 & 0.8633 & 0.6953 & 0.9092 & 1.0000
\end{bmatrix}$$

$\gamma^{(P)} =$

$$\begin{bmatrix}
1.0000 & 0.7717 & 0.8184 & 0.7633 & 0.8644 & 0.9198 & 0.9089 & 0.8588 & 0.8697 & 0.8381 \\
0.9262 & 1.0000 & 0.7196 & 0.7353 & 0.9588 & 0.9840 & 0.8463 & 0.7853 & 0.7648 & 0.7666 \\
0.7936 & 0.8113 & 1.0000 & 0.7441 & 0.8513 & 0.9274 & 0.7042 & 0.8250 & 0.8528 & 0.7291 \\
0.7977 & 0.8136 & 0.9174 & 1.0000 & 0.8501 & 0.7205 & 0.8204 & 0.7631 & 0.8086 & 0.8773 \\
0.8256 & 0.7828 & 1.0000 & 0.7830 & 1.0000 & 0.7872 & 0.8519 & 0.7275 & 0.7577 & 0.8263 \\
0.8329 & 0.7944 & 0.5845 & 0.8226 & 0.7242 & 1.0000 & 0.8258 & 0.8666 & 0.8125 & 0.6453 \\
0.7948 & 0.6633 & 0.6590 & 0.7301 & 0.7309 & 0.7954 & 1.0000 & 0.6924 & 1.0000 & 0.7572 \\
0.6132 & 0.8242 & 0.9830 & 0.8946 & 0.9385 & 0.8337 & 0.6134 & 1.0000 & 0.7439 & 0.9050 \\
1.0000 & 0.8272 & 1.0000 & 0.7849 & 0.8794 & 0.8221 & 0.8042 & 0.8693 & 1.0000 & 0.9213 \\
0.6377 & 0.8034 & 0.8878 & 0.7836 & 0.8224 & 0.8371 & 0.8678 & 0.8240 & 0.8049 & 1.0000
\end{bmatrix}$$

$r^{(P)} = -0.1$

The bifurcation parameter $r^{(A)}$ changes linearly in in 50 steps from -0.0424 to -1.2106 for all pollinators, chosen in such a way that the dominant eigenvalue of the Jacobian matrix changes from -0.45 to -0.15 as the system approaches a critical transition (supplementary fig. 1).

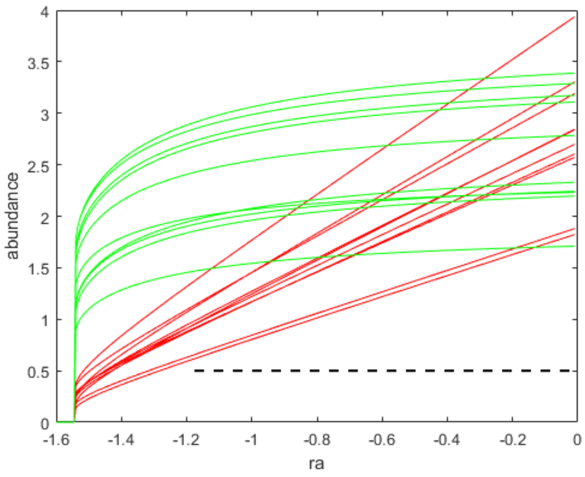

Figure 1: Behaviour of the 20D model without noise with a full network collapse. Green lines indicate plant abundances and red lines indicate pollinator abundances. The dashed black line depicts the values for $r^{(A)}$ that we use for our analysis.

2 parameter settings of the 20D model with partial network collapse

$c^{(A)} =$

$$\begin{bmatrix} 0.3000 & 0.0566 & 0.0525 & 0.0181 & 0.0516 & 0.0286 & 0.0510 & 0.0510 & 0.0635 & 0.0271 \\ 0.0517 & 0.3000 & 0.0724 & 0.0255 & 0.0508 & 0.0317 & 0.0358 & 0.0216 & 0.0444 & 0.0194 \\ 0.0085 & 0.0427 & 0.3000 & 0.0183 & 0.0235 & 0.0615 & 0.0009 & 0.0363 & 0.0468 & 0.0283 \\ 0.0418 & 0.0431 & 0.0361 & 0.3000 & 0.0572 & 0.0134 & 0.0524 & 0.0481 & 0.0603 & 0.0184 \\ 0.0421 & 0.0233 & 0.0480 & 0.0555 & 0.3000 & 0.0097 & 0.0220 & 0.0550 & 0.0407 & 0.0331 \\ 0.0381 & 0.0481 & 0.0158 & 0.0485 & 0.0583 & 0.3000 & 0.0480 & 0.0286 & 0.0388 & 0.0181 \\ 0 & 0.0608 & 0.0324 & 0.0282 & 0.0298 & 0.0629 & 0.3000 & 0.0200 & 0.0543 & 0.0451 \\ 0.0273 & 0.0594 & 0.0258 & 0.0364 & 0.0591 & 0.0148 & 0.0119 & 0.3000 & 0.0237 & 0.0559 \\ 0.0519 & 0.0439 & 0.0403 & 0.0210 & 0.0445 & 0.0274 & 0.0481 & 0.0243 & 0.3000 & 0.0070 \\ 0.0332 & 0.0069 & 0.0347 & 0.0470 & 0.0101 & 0.0496 & 0.0309 & 0.0264 & 0.0234 & 0.3000 \end{bmatrix}$$

$c^{(P)} =$

$$\begin{bmatrix} 0.3000 & 0.0723 & 0.0379 & 0.0616 & 0.0127 & 0.0531 & 0.0275 & 0.0421 & 0.0248 & 0.0378 \\ 0.0460 & 0.3000 & 0.0038 & 0.0612 & 0.0664 & 0.0333 & 0.0293 & 0.0449 & 0.0360 & 0.0020 \\ 0.0344 & 0.0320 & 0.3000 & 0.0374 & 0.0585 & 0.0582 & 0.0680 & 0.0382 & 0.0676 & 0.0128 \\ 0.0184 & 0.0228 & 0.0371 & 0.3000 & 0.0392 & 0.0627 & 0.0575 & 0.0208 & 0.0461 & 0.0283 \\ 0.0402 & 0.0470 & 0.0392 & 0.0435 & 0.3000 & 0.0495 & 0.0195 & 0.0429 & 0.0591 & 0.0340 \\ 0.0431 & 0.0520 & 0.0581 & 0.0718 & 0.0269 & 0.3000 & 0.0079 & 0.0379 & 0.0408 & 0.0534 \\ 0.0615 & 0.0129 & 0.0262 & 0.0431 & 0.0396 & 0.0186 & 0.3000 & 0.0475 & 0.0253 & 0.0425 \\ 0.0461 & 0.0583 & 0.0134 & 0.0490 & 0.0126 & 0.0216 & 0.0446 & 0.3000 & 0.0577 & 0.0657 \\ 0.0290 & 0.0306 & 0.0290 & 0.0600 & 0.0147 & 0.0692 & 0.0807 & 0.0492 & 0.3000 & 0.0211 \\ 0.0345 & 0.0322 & 0.0443 & 0.0193 & 0.0409 & 0.0349 & 0.0504 & 0.0506 & 0.0441 & 0.3000 \end{bmatrix}$$

$\gamma^{(A)} =$

$$\begin{bmatrix} 1.0000 & 0.8529 & 0.7304 & 0.9240 & 0.6789 & 0.8022 & 0.6468 & 0.5195 & 0.9032 & 0.8485 \\ 0.7804 & 1.0000 & 0.8725 & 0.7045 & 0.8580 & 0.7597 & 0.8700 & 0.6865 & 0.8380 & 0.6699 \\ 0.8359 & 0.6892 & 1.0000 & 0.7063 & 0.7855 & 0.7843 & 0.6484 & 0.9288 & 0.7659 & 0.8341 \\ 0.7470 & 0.7224 & 0.9097 & 1.0000 & 0.7419 & 0.6929 & 0.8887 & 0.7431 & 0.9261 & 0.8208 \\ 0.7901 & 0.6600 & 0.9390 & 0.8583 & 1.0000 & 0.9169 & 0.6562 & 0.8008 & 0.8312 & 0.7562 \\ 0.6107 & 0.8838 & 0.7761 & 0.6947 & 0.8547 & 1.0000 & 0.8929 & 0.6877 & 0.7465 & 0.7407 \\ 0.7592 & 0.8284 & 0.6102 & 0.7943 & 0.8421 & 0.8563 & 1.0000 & 0.8955 & 0.8385 & 0.8438 \\ 0.7883 & 0.8154 & 0.8696 & 0.8296 & 0.8414 & 0.8169 & 0.6932 & 1.0000 & 0.7579 & 0.8582 \\ 0.6963 & 0.7577 & 1.0000 & 0.7533 & 0.8661 & 0.9028 & 0.8552 & 0.7388 & 1.0000 & 0.7857 \\ 0.7951 & 0.7947 & 0.6607 & 0.8321 & 0.7693 & 0.8694 & 0.7682 & 0.6940 & 0.7994 & 1.0000 \end{bmatrix}$$

$\gamma^{(P)} =$

$$\begin{bmatrix}
1.0000 & 1.0000 & 0.7609 & 0.7574 & 0.8573 & 0.8231 & 0.6850 & 0.7173 & 0.8598 & 0.8089 \\
0.8550 & 1.0000 & 0.8879 & 0.7032 & 0.9633 & 0.8709 & 0.8280 & 0.8565 & 0.9481 & 0.7359 \\
0.6711 & 0.7216 & 1.0000 & 0.7861 & 0.7660 & 0.7909 & 0.8975 & 0.7717 & 0.7193 & 0.7823 \\
0.6764 & 0.7527 & 0.9938 & 1.0000 & 0.7275 & 0.6884 & 0.8215 & 0.7282 & 0.7369 & 0.6859 \\
0.7902 & 0.7127 & 1.0000 & 0.9249 & 1.0000 & 0.9663 & 0.7082 & 0.8111 & 0.6957 & 0.8916 \\
0.9422 & 0.7436 & 0.8804 & 0.8826 & 0.8622 & 1.0000 & 0.8426 & 0.8001 & 0.7651 & 0.8607 \\
0.8188 & 0.6296 & 0.7034 & 0.8614 & 0.8303 & 0.7966 & 1.0000 & 0.7652 & 0.9753 & 0.7626 \\
0.9492 & 0.7197 & 0.7458 & 0.7138 & 0.6965 & 0.9203 & 0.9097 & 1.0000 & 0.6572 & 0.6437 \\
0.6836 & 0.9059 & 0.8163 & 0.7971 & 0.6762 & 0.9395 & 0.7365 & 0.7183 & 1.0000 & 0.8436 \\
0.7899 & 0.6714 & 0.9072 & 0.5238 & 0.8976 & 0.7449 & 0.8339 & 0.7834 & 0.7596 & 1.0000
\end{bmatrix}$$

$r^{(P)} = -0.1$

The bifurcation parameter $r^{(A)}$ changes linearly in in 50 steps from -0.4318 to -0.8537 for the first 5 pollinators. For the other pollinators it is set to -0.4318 and it remains there. These values are chosen in such a way that the dominant eigenvalue of the Jacobian matrix changes from -0.45 to -0.15 as the system approaches a critical transition.

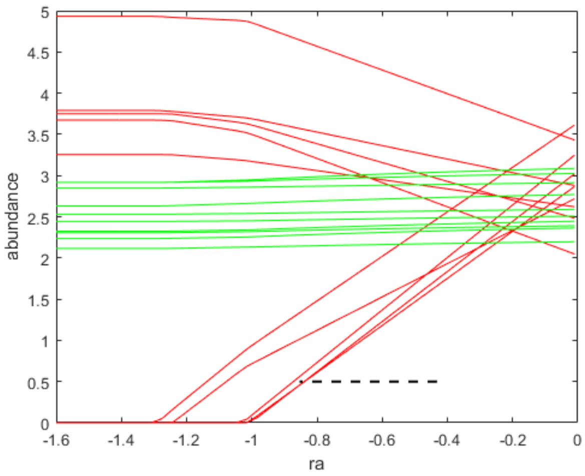

Figure 2: Behaviour of the 20D model without noise with a partial network collapse. Green lines indicate plant abundances and red lines indicate pollinator abundances. The dashed black line depicts the values for $r^{(A)}$ that we use for our analysis.

3 supplementary figures

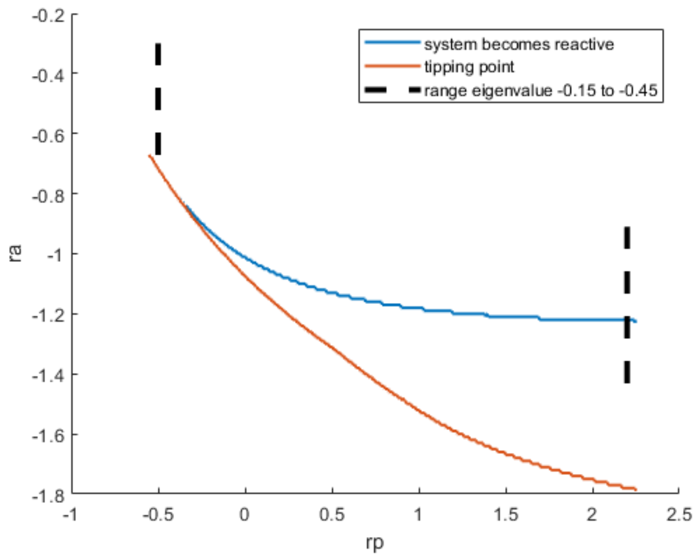

Figure 3: Illustration of the combination of values for $r^{(A)}$ (here denoted ra) and $r^{(P)}$ (here rp) where the 4 dimensional plant-pollinator system becomes reactive and where the tipping point is located. Dashed lines indicate the range of $r^{(A)}$ that we use for our analysis where we push the system towards the tipping point, chosen in such a way that the dominant eigevalue of the Jacobian ranges from -0.45 to -0.15.

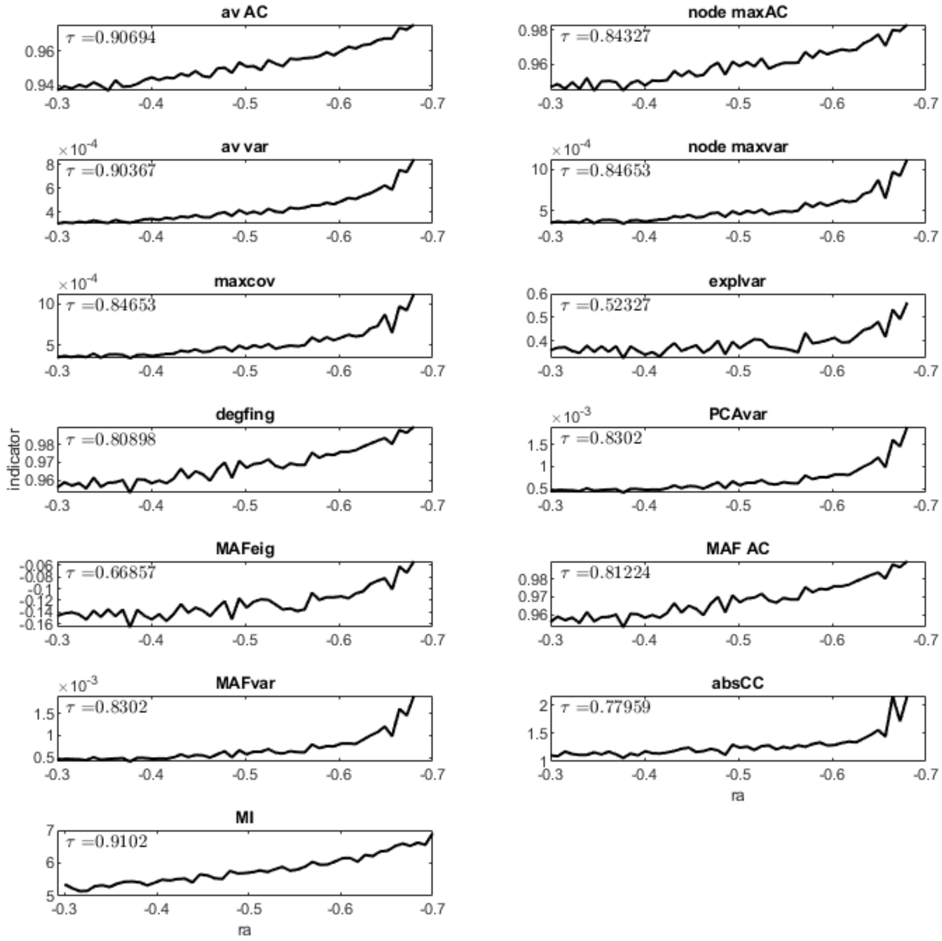

Figure 4: Illustration of the increase in all of the indicators in the 4D model as the bifurcation parameter $r^{(A)}$ (here denoted ra) reaches the critical value. For every value of ra, 10,000 time points are generated. The Kendall tau correlation for the trend is indicated in every subplot.

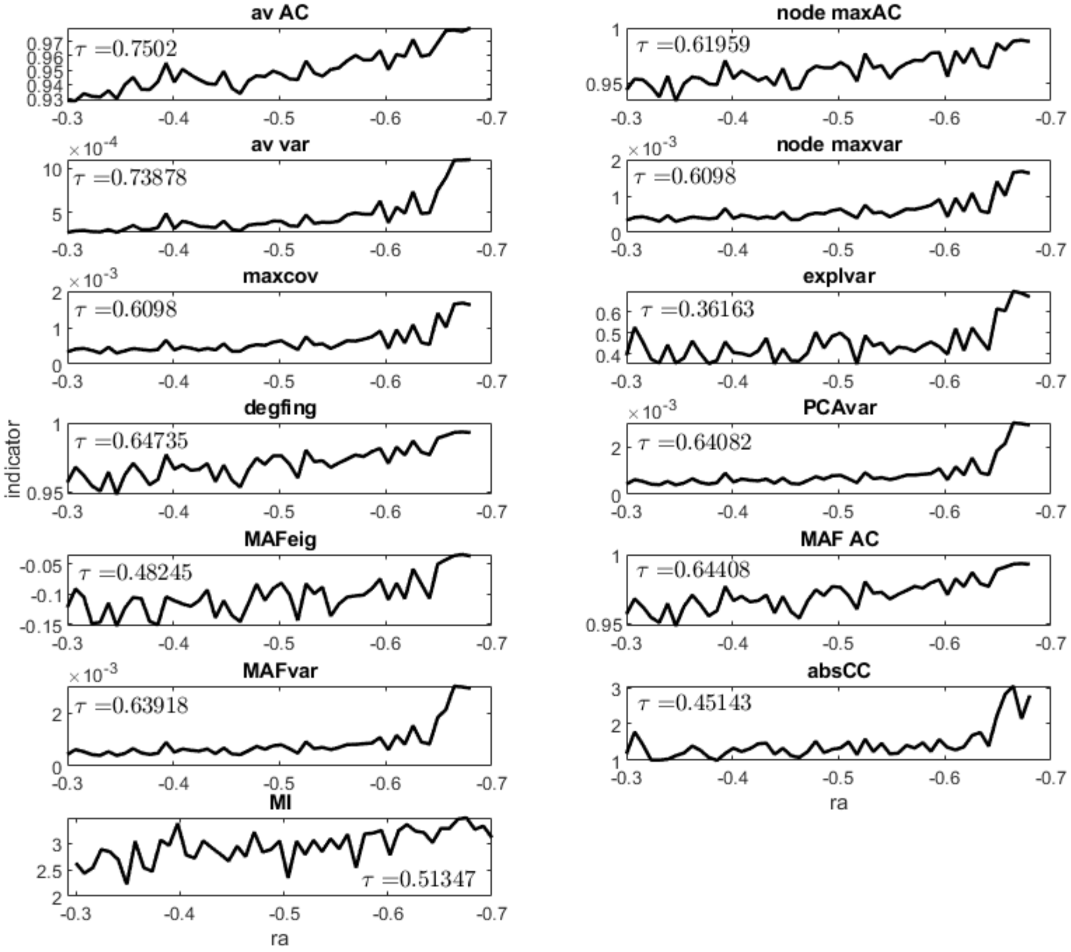

Figure 5: Same as figure S4, but with time series with a length of 1000 instead of 10,000 per value for ra, yielding a lower performance than with 10,000 points.

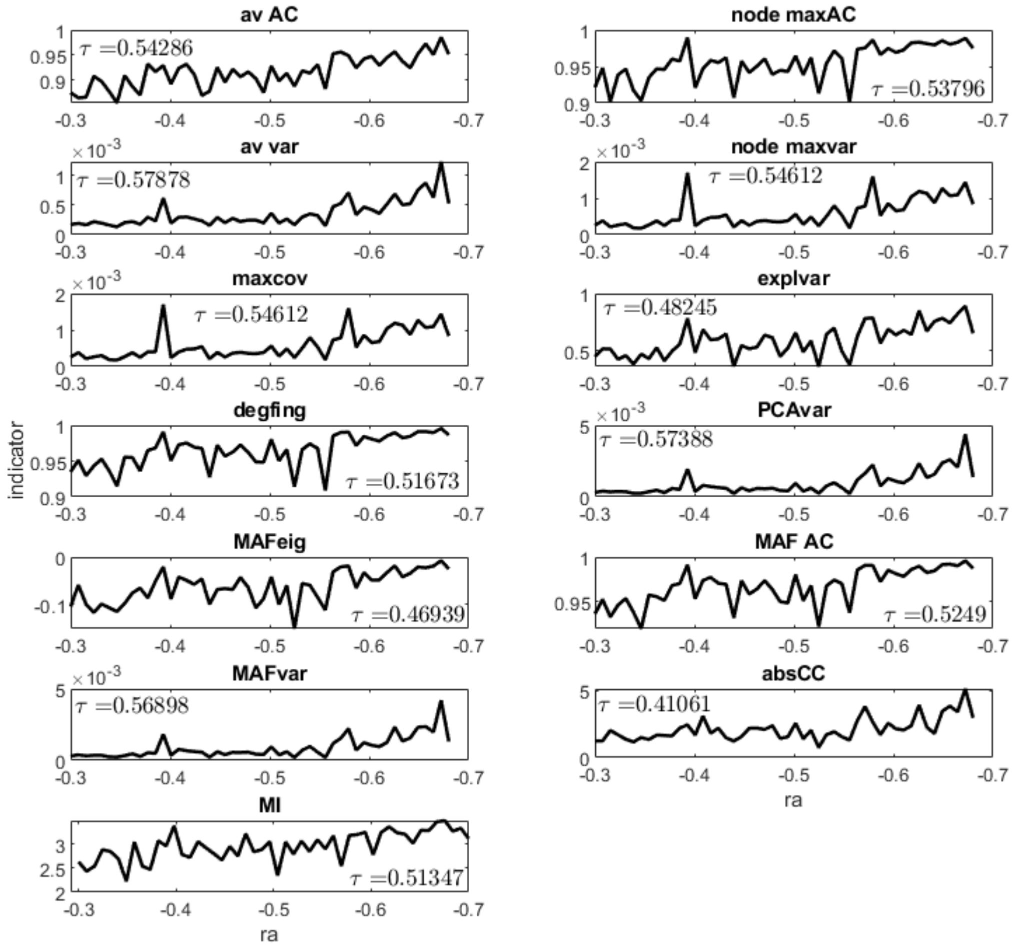

Figure 6: Same as figure S4, but with time series with a length of 100 instead of 10,000 per value for ra, yielding a lower performance than with 10,000 or 1000 points.

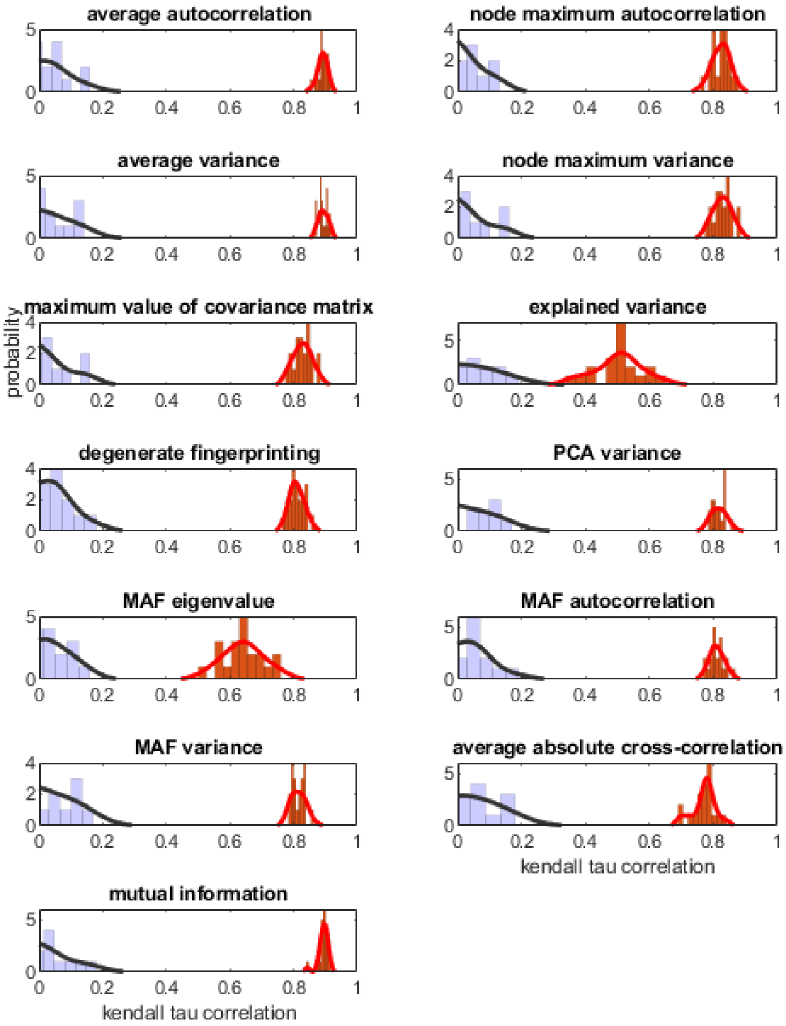

Figure 7: Distributions of 20 simulations for the 4-dimension model. The blue distributions are Kendall tau correlations for different indicators for time series that do not change over time (the parameter $r^{(A)}$ is fixed). In this situation, no change is expected and thus the Kendall tau correlation is centered around zero. The red distributions are distributions of the Kendall tau correlations for data that is pushed towards a tipping point (for one instance, see figure 4). This figure shows that the distributions do not overlap and for this particular data set the specificity of the Kendall tau correlation is high, suggesting a low rate of false positives.

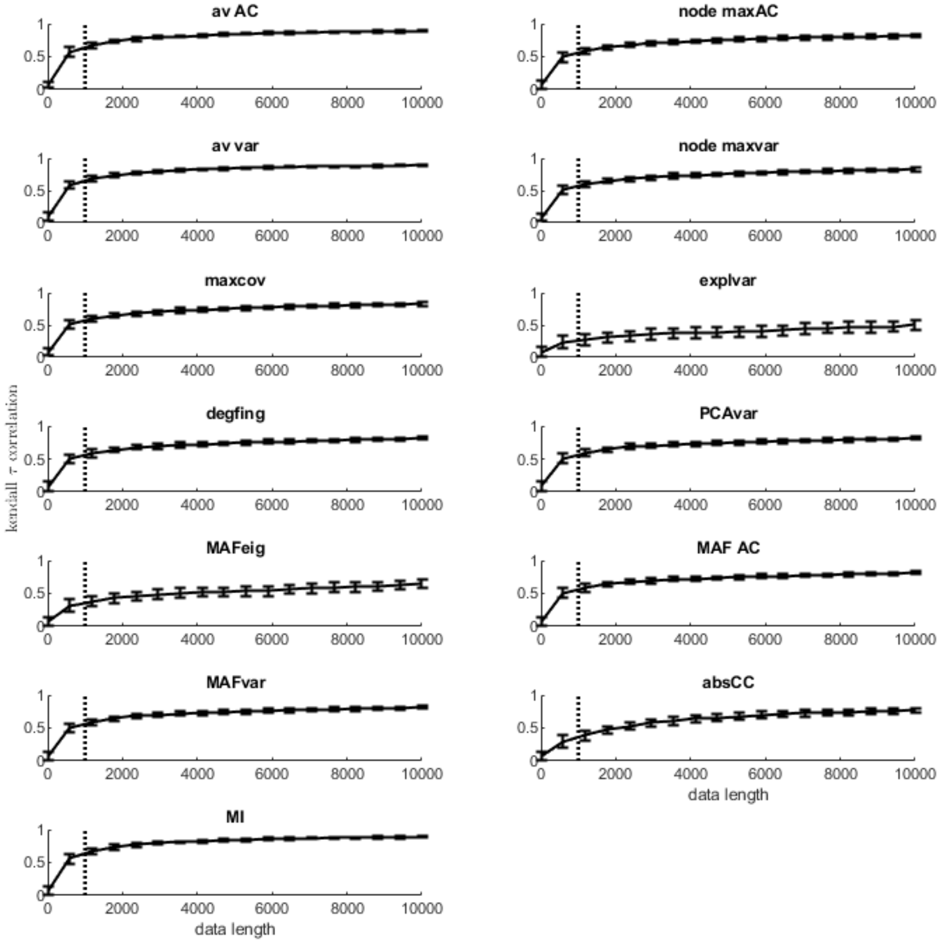

Figure 8: The effect of different data lengths for the analyses. Lines demonstrate the increase in kendall tau correlation as the data length increases. Error bars indicate standard deviations of 20 different simulations. The dotted line indicates the data length used for our 'reduced data length scenario'. All other analyses were performed on the largest data size indicated here (10.000 time points).

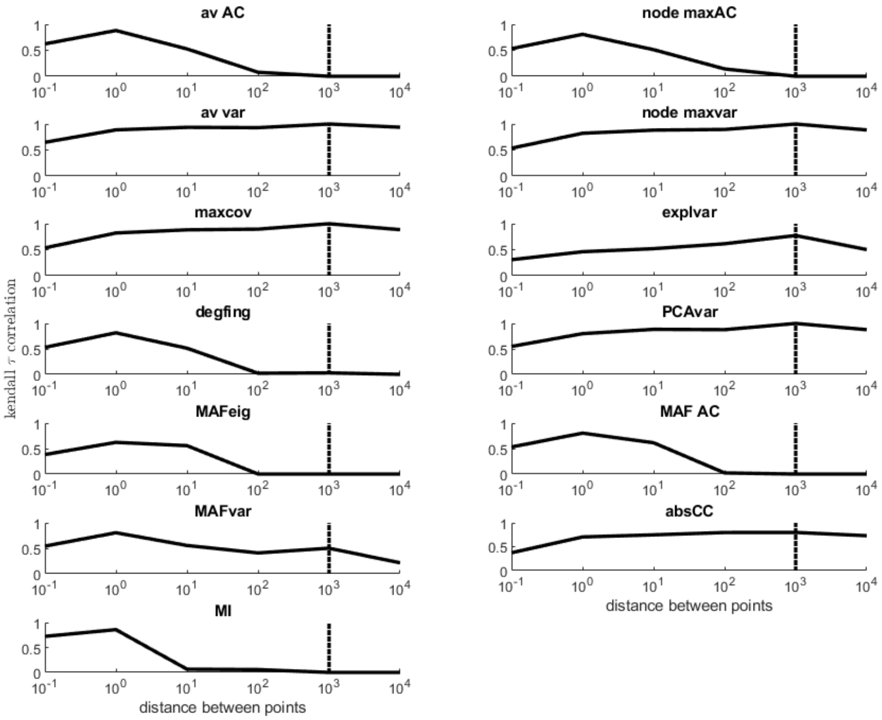

Figure 9: The effect of different data resolutions for the analyses with data with a length of 1000. Lines demonstrate the increase or decrease in kendall tau correlation as the data length increases. The dotted line indicates the data length used for our 'reduced data resolution scenario'. All other analyses were performed on the highest data resolution indicated here (a distance of 10^{-1} time points).

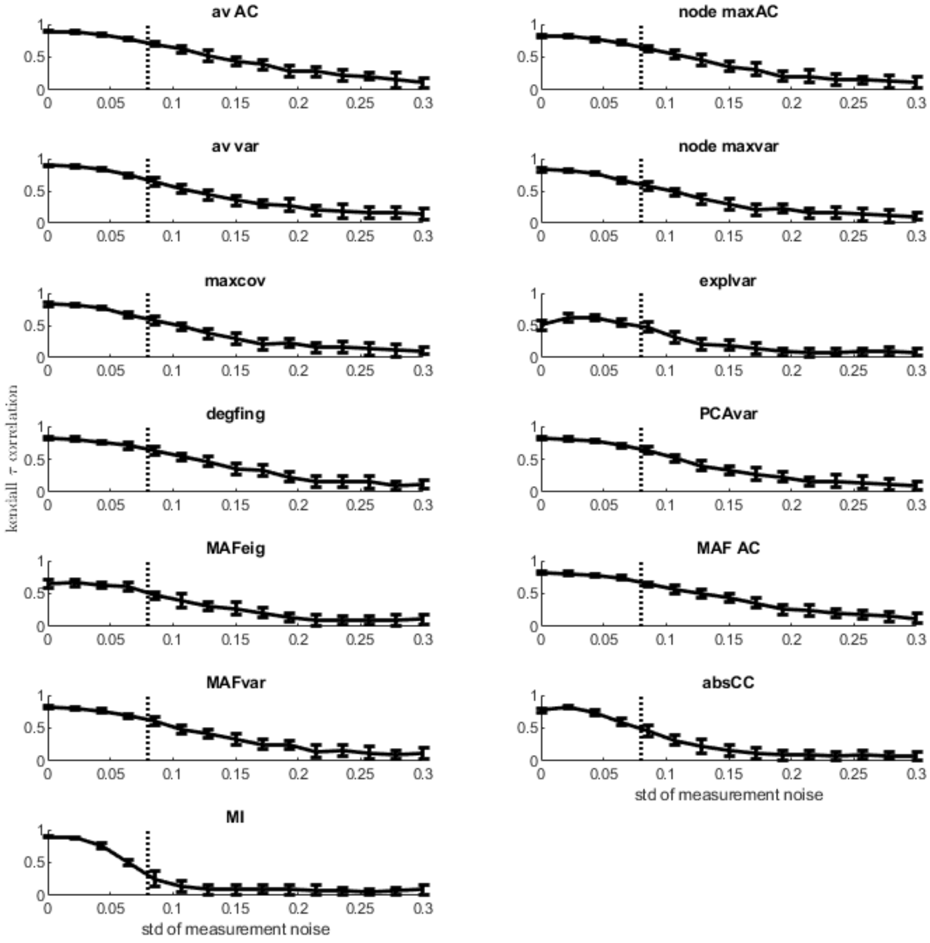

Figure 10: The effect of different measurement noise or observation noise for the analyses. Lines demonstrate the decrease in kendall tau correlation as the standard deviation of the measurement noise increases. Error bars indicate standard deviations of 20 different simulations. The dotted line indicates the measurement noise used for our 'measurement noise scenario'. All other analyses were performed without measurement noise.

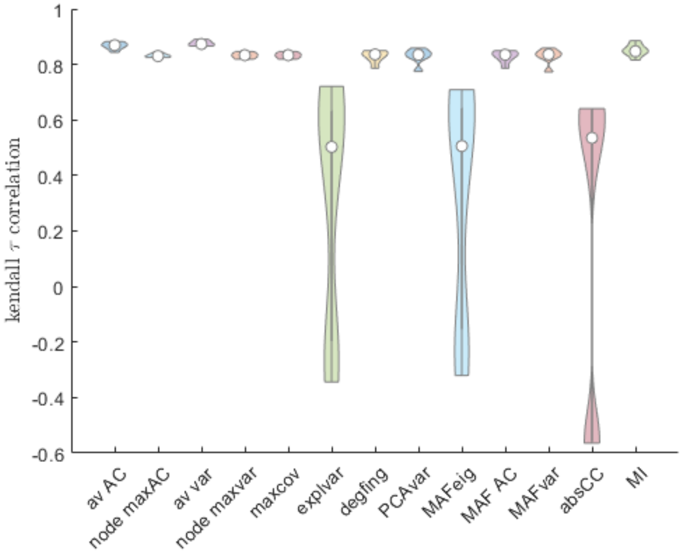

Figure 11: Violin plots of the distributions of Kendall τ correlations of all 6 possibilities of taking 2 out of the 4 variables in the 4D model. White circle indicates the median value. The lowest values are used as the indicator's performance in figure 1, subset worst case in the main text, while the highest values are used for the subset best case. Kendall τ correlations lower than zero indicate a reversed effect, i.e. the indicator predicts an increase in resilience as the system moves towards the tipping point.

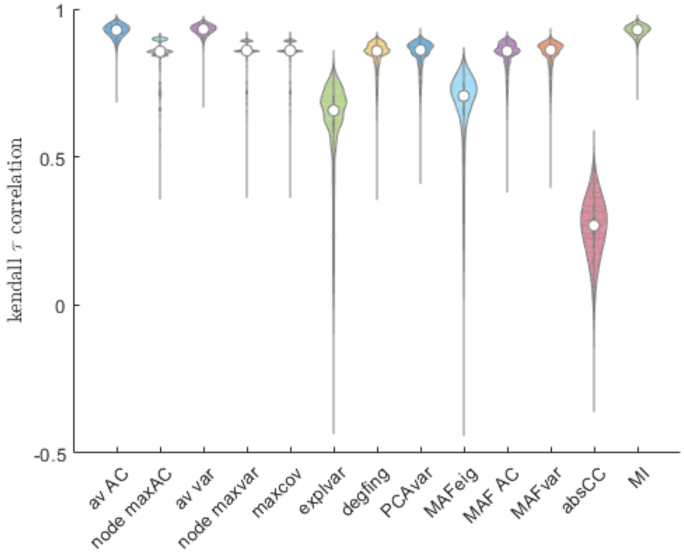

Figure 12: Violin plots of the distributions of Kendall τ correlations of all 184765 possibilities of taking 10 out of the 20 variables in the 20D model with a full network collapse. White circle indicates the median value. The 5% and 95% quantiles are used as the 'subset - worst case' and 'subset - best case' in figure 2 in the main text. Kendall τ correlations lower than zero indicate a reversed effect, i.e. the indicator predicts an increase in resilience as the system moves towards the tipping point.

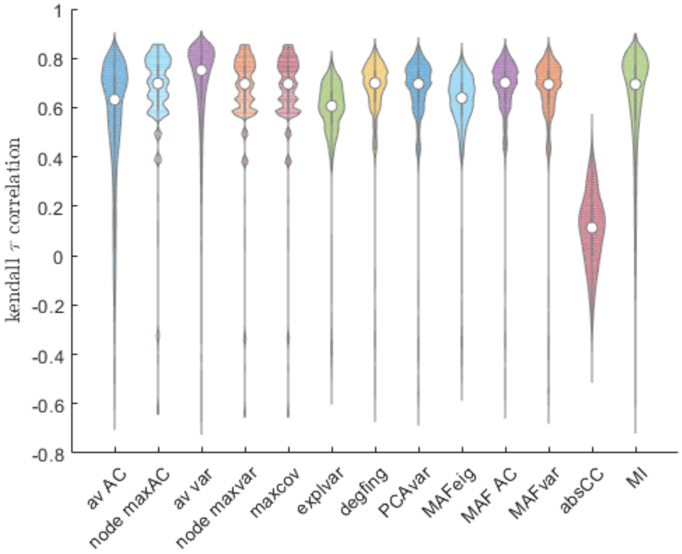

Figure 13: Violin plots of the distributions of Kendall τ correlations of all 184765 possibilities of taking 10 out of the 20 variables in the 20D model with a partial network collapse. White circle indicates the median value. The 5% and 95% quantiles are used as the 'subset - worst case' and 'subset - best case' in figure 3 in the main text. Kendall τ correlations lower than zero indicate a reversed effect, i.e. the indicator predicts an increase in resilience as the system moves towards the tipping point.

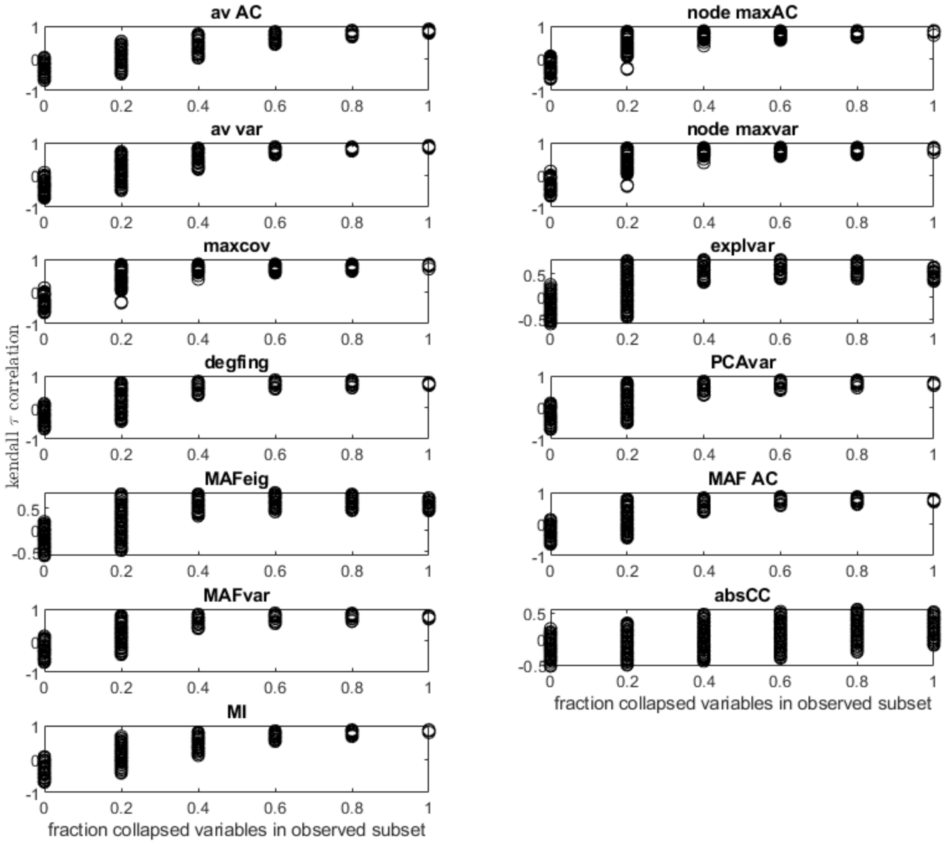

Figure 14: Kendall τ correlations per indicator for all simulations where a fraction of the collapsed variables are present. A fraction of 0 indicates that none of the collapsing variables are within the subset of observed variables and a fraction of 1 indicates that all collapsing variables are within the subset of observed variables. Highest performance is obtained when all collapsed variables are observed.
